# Metabolic Discrimination between Adventitious Roots and Standard Medicinal Part of *Atractylodes macrocephala* Koidz. Using FT-IR Spectroscopy

**DOI:** 10.3390/plants12091821

**Published:** 2023-04-28

**Authors:** So Yeon Choi, Seong Sub Ku, Myung Suk Ahn, Eun Jin So, HyeRan Kim, Sang Un Park, Moon-Soon Lee, Young Min Kang, Sung Ran Min, Suk Weon Kim

**Affiliations:** 1 Plant Systems Engineering Research Center, Korea Research Institute of Bioscience and Biotechnology (KRIBB), 125 Gwahak-ro, Yuseong–gu, Daejeon 34141, Republic of Korea; 2 Department of Crop Science, Chungnam National University, 99 Daehak-ro, Yuseong-gu, Daejeon 34134, Republic of Korea; 3 Department of Industrial Plant Science and Technology, Chungbuk National University, 1 Cheongdae-ro, Seowon-gu, Cheongju 28644, Republic of Korea; 4 Floriculture Research Division, National Institute of Horticultural and Herbal Science, RDA, Wanju 55365, Republic of Korea; 5 Herbal Medicine Resources Research Center, Korea Institute of Oriental Medicine (KIOM), 111 Geonjae-ro, Naju 58245, Republic of Korea; 6 Biological Resources Center, Korea Research Institute of Bioscience and Biotechnology, 181 Ipsingil, Jeongeup 56212, Republic of Korea

**Keywords:** indole-3-butyric acid (IBA), liquid culture, medicinal plants, partial least square discriminant analysis (PLS-DA), principal component analysis (PCA)

## Abstract

This study aims to examine the metabolic discrimination between in vitro grown adventitious roots and the standard medicinal parts of *Atractylodes macrocephala*. To achieve this goal, firstly, in vitro culture conditions of adventitious roots such as indole-3-butyric acid (IBA) concentrations, types of media, inorganic salt strength of culture medium, and elicitor types and concentrations were optimized. The optimal culture conditions for proliferation of adventitious roots was found to consist of Murashige and Skoog (MS) medium containing 5 mg L^−1^ IBA. Whole cell extracts from adventitious roots and the standard medicinal parts of *A. macrocephala* were subjected to Fourier transform infrared spectroscopy (FT-IR). Principal component analysis (PCA) and partial least square discriminant analysis (PLS-DA) from FT-IR spectral data showed that adventitious roots and standard medicinal parts were clearly distinguished in the PCA and PLS-DA score plot. Furthermore, the overall metabolite pattern from adventitious roots was changed depending on the dose-dependent manner of chemicals. These results suggest that FT-IR spectroscopy can be applied as an alternative tool for the screening of higher metabolic root lines and for discriminating metabolic similarity between in vitro grown adventitious roots and the standard medicinal parts. In addition, the adventitious roots proliferation system established in this study can be directly applied as an alternative means for the commercial production of *A. macrocephala.*

## 1. Introduction

Baekchul is a rhizome of *Atractylodes macrocephala* Koidz. or *Atractylodes japonica* Koidz., a perennial herbaceous plant belonging to the Asteraceae family. It is a medicinal plant widely used in East Asia including Korea, China, and Japan [1]. Over 200 chemical compounds have been isolated from *Atractylodis* Rhizoma (AR) including a series of sesquiterpenoids, alkynes, triterpenoids, aromatic glycosides, oligosaccharides, and polysaccharides [2,3,4]. In particular, *A. macrocephala* contains volatile components in its essential oil, and the main volatile components are sesquiterpenoids such as atractylenolide (I, II, and III), actractylon, 3β-acetoxyatractylone, and 3β-hydroxyatractylone [5]. Although, recent research has revealed that the extracts from AR could help to improve gastrointestinal function, anti-tumor activity, immunomodulatory activity, anti-inflammatory activity, and anti-bacterial activity [4]. However, the toxicity and safety of the chemicals have not been fully defined [6].

Despite the valuable pharmacological properties of AR, *A. japonica* presents several horticultural challenges that limit potential for the mass production of the medicinal rhizome. Most significantly, it has a low seed set rate and a slow growth rate for its rhizomes [7]. Baekchul (*A. macrocephala*) is also difficult to cultivate due to low resistance to root rot disease [8]. Therefore, the development of novel approaches for the mass production of medicinal AR is an indispensable topic. Plant cell and tissue culture systems could be applied as a novel approach for the mass supply of medicinal plant resources under aseptic culture conditions. In particular, adventitious roots proliferated in vitro offer an attractive source for useful phytochemicals due to their genetic stability and biosynthetic capacity [9]. Adventitious roots can be easily induced from several differentiated plant organs, such as the leaf, stem, and root.

To date, tissue culture studies of *A. macrocephala* have mainly focused on the plant regeneration system, including clonal multiplication of *Atractylodes lancea* by shoot tip culture [10] and the effect of plant growth regulators on plant regeneration from the auxiliary bud of the *Atractylodes* species [1,11]. However, there are only a few studies published that treat the establishment of a hairy roots culture system using leaf explants of *A. japonica* [12]. There has not been a published study of the induction and mass propagation system of adventitious roots from *A. macrocephala*.

To employ the in vitro proliferated adventitious roots as a herbal medicine resource, it is necessary to investigate differences in quantitative and qualitative patterns. In order to compare metabolic similarity between standard medicinal parts of *A. macrocephala* and their adventitious roots, the metabolite fingerprinting approach using 1HNMR (proton nuclear magnetic resonance spectroscopy), mass spectrometry, and FT-IR (Fourier transform infrared spectroscopy) is very efficient [13,14]. In particular, FT-IR spectroscopy, combined with multivariate statistical analysis from whole plant cell extracts, can be applied with high reproducibility and sensitivity. FT-IR spectral analysis is widely used to identify closely related microbial species [15,16], for the classification of plant species [17], and for cultivar identification [18,19]. Moreover, FT-IR spectral analysis can be applied in the comparison of the metabolic similarity between standard medicinal parts and their in vitro proliferated adventitious roots [20,21].

Therefore, we attempted to establish the optimal culture conditions for the mass production of adventitious roots of *A. macrocephala* in this study. First, we investigated the effects of IBA concentrations, the impact of types of media, inorganic salt concentrations, and elicitor types and concentrations on the growth of adventitious roots. Secondly, a quantitative analysis system using HPLC analysis to investigate the content of active compounds between adventitious roots after elicitor treatment and standard roots. Furthermore, a rapid metabolic discrimination system was developed using FT-IR spectroscopy for the investigation of the metabolic equivalence between in vitro grown adventitious roots and standard medicinal parts.

## 2. Results

### 2.1. Effect of IBA Concentrations on Growth of Adventitious Roots

**Figure 1:**
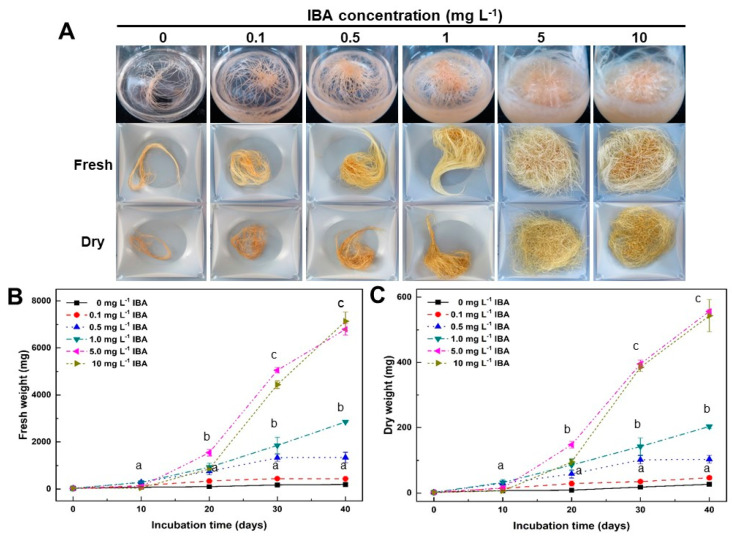
Effect of IBA concentrations on adventitious root growth of *A. macrocephala* after 40 days of culture in Murashige and Skoog medium containing various concentrations of IBA. (**A**) Root morphology. (**B**) Change in fresh weight of adventitious roots. (**C**) Change in dry weight of adventitious roots. Results are expressed as mean ± SD (n = 3). Different superscript letters indicate a significant difference (*p* < 0.05).

In order to investigate the effect of the optimal IBA concentration on the growth of adventitious roots, adventitious roots were transferred to MS [22] medium containing 30 g L^−1^ sucrose and several concentrations of IBA (Figure 1A). After 10 days of culture, longitudinal growth and root branching from inoculated adventitious roots began to be seen in most IBA treatments except for those subjected to treatment of lower than 0.1 mg L^−1^ IBA. In the 5–10 mg L^−1^ IBA treatments, adventitious roots rapidly proliferated from the 20th to 40th days of culture (Figure 1B,C). After 40 days of culture, the changes in fresh and dry weight from adventitious roots were examined. The fresh weight from dventitious roots was the highest at 7136.7 ± 395.5 mg (Figure 1B) in the 10 mg L^−1^ IBA treatment. Additionally, the dry weight from adventitious roots was the highest at 556.7 ± 5.8 mg in 5 mg L^−1^ IBA treatment (Figure 1C). Overall, there was no significant difference in the growth rate of adventitious roots between those subjected to the 5 mg L^−1^ IBA and the 10 mg L^−1^ IBA treatments. However, overall, the increase in fresh and dry weight from adventitious roots appeared to be dependent on the IBA concentration. In light of these results, the optimum concentration of IBA required for adventitious root proliferation of *A. macrocephala* was determined to be 5 mg L^−1^ IBA. In subsequent experiments, the IBA was set at a concentration of 5 mg L^−1^.

The analysis of the FT-IR spectral data, combined with multivariate analysis of the adventitious roots treated with IBA concentrations and the standard medicinal parts of *A. macrocephala*, revealed that there had been a quantitative and qualitative change between samples in the 1700–1500, 1450–1200, and 1100–900 cm^−1^ regions of FT-IR spectra (see Supplementary Materials, Figure S1A,C). The PCA and PLS-DA score plot also showed that adventitious roots treated with IBA concentrations and the standard medicinal parts of *A. macrocephala* could be clearly discriminated (see Supplementary Materials, Figure S1B,D). These results clearly confirm that there is a significant difference between the adventitious roots and the standard medicinal parts of *A. macrocephala* at the whole metabolite level. Furthermore, these results also reveal that the whole metabolite pattern of adventitious roots changes in accord with the increase in the concentration of IBA.

### 2.2. Effect of Culture Media and Inorganic Salt Concentrations in MS Medium on Growth of Adventitious Roots

**Figure 2:**
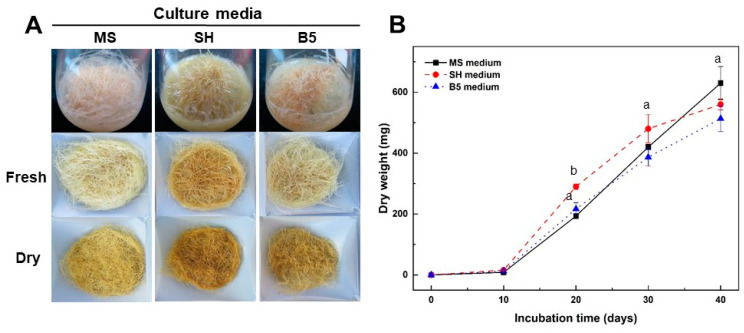
Effect of culture media on adventitious root growth of *A. macrocephala* after 40 days of culture in various media containing 5 mg L^−1^ IBA. (**A**) Root morphology. (**B**) Change in dry weight of adventitious roots. Results are expressed as mean ± SD (n = 3). Different superscript letters indicate a significant difference (*p* < 0.05).

**Figure 3:**
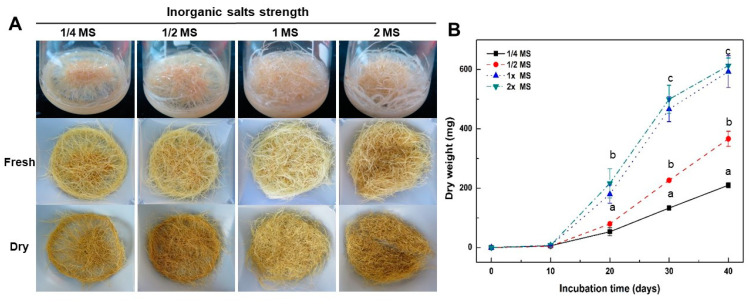
Effect of inorganic salt concentrations on adventitious root growth of A. macrocephala after 40 days of culture in 1/4, 1/2, 1, and 2x MS inorganic salt medium containing 5 mg L^−1^ IBA. (**A**) Root morphology. (**B**) Change in dry weight of adventitious roots. Results are expressed as mean ± SD (n = 3). Different superscript letters indicate a significant difference (*p* < 0.05).

The effect of culture media and inorganic salt concentrations in MS medium on the growth of adventitious roots was examined (Figure 2 and Figure 3). As part of the investigation of the effect of culture media on growth of adventitious roots, roots were transferred to MS, Schenk and Hildebrandt (SH) [23], and Gamborg et al. (B5) [24] media containing 5 mg L^−1^ IBA (Figure 2). After 10 days of culture, the adventitious roots cultured in SH and B5 medium began to brown slightly more than those in MS medium. However, adventitious roots continued to grow vigorously until 40 days of culture in all culture media (Figure 2A). After 40 days of culture, the change in the dry weight of the adventitious roots was examined. The dry weight from adventitious roots was the highest at 630 ± 54.4 mg in MS medium (Figure 2B). However, there was no significant difference in the growth rate of adventitious roots from that observed in culture media types. Considering these results, the optimal culture media required for adventitious root proliferation of *A. macrocephala* was determined to be the MS medium. In the subsequent experiments, the MS medium was used to encourage adventitious root proliferation.

Whole cell extracts were subjected to FT-IR spectroscopy (see Supplementary Materials, Figure S2) so as to compare the overall metabolic changes from the adventitious roots cultured on three different culture media and the standard medicinal parts of *A. macrocephala*. Major spectral changes were observed in the 1700–1500, 1450–1200, and 1100–900 cm^−1^ regions of FT-IR spectra (see Supplementary Materials, Figure S2A,C). The PCA and PLS-DA score plot also revealed that adventitious roots and the standard medicinal parts were clearly discriminated (see Supplementary Materials, Figure S2B,D). These results suggest that there is a significant difference between adventitious roots and standard medicinal parts at the whole metabolite level. Furthermore, these results also show that the whole metabolite pattern of adventitious roots from the SH medium is more similar to the B5 medium than to the MS medium.

To investigate the effect of the concentration of inorganic salts in the MS medium on the growth of adventitious roots, roots were transferred to a medium of 1/4, 1/2, 1, and 2 times strength of MS inorganic salts containing 5 mg L^−1^ IBA and 30 g L^−1^ sucrose (Figure 3). After 10 days in the culture, the adventitious roots cultured in low concentrations (1/4 and 1/2) of MS inorganic salts did not branch lateral roots. In particular, adventitious roots did not proliferate in the 1/4 strength MS inorganic salts medium. After 20 days in the culture, lateral roots were induced from adventitious roots into all treatments. Lateral root development from the adventitious roots increased in accord with increasing MS inorganic salt concentrations. However, the proliferation of adventitious roots in the 1- and 2-times strength MS medium was much higher than that in the low concentration (1/4 and 1/2) MS medium for the first 40 days of culture (Figure 3A). After 40 days of culture, the change in dry weight from adventitious roots was examined. The dry weight from adventitious roots was the highest at 613.3 ± 25.8 mg in 2x MS medium (Figure 3B). However, there was no significant difference in the increase in dry weight in the 1x MS medium. These results suggest that the optimal inorganic salt concentration for adventitious root proliferation of A. macrocephala should be 1x MS medium. In the subsequent experiments, the 1x MS medium was used to promote adventitious root proliferation.

Similar to FT-IR analysis of IBA concentrations, FT-IR based metabolic changes of adventitious roots in MS inorganic salt concentration treatments revealed the same concentration dependency manner (see Supplementary Materials, Figure S3). The PCA and PLS-DA score plot showed that adventitious roots treated with MS inorganic salt concentrations and the standard medicinal parts of *A. macrocephala* were clearly discriminated (see Supplementary Materials, Figure S3B,D). These results clearly reveal a significant difference between adventitious roots and standard medicinal parts of *A. macrocephala* at the whole metabolite level. Furthermore, these results also show that the whole metabolite pattern for adventitious roots changes as the concentration of MS inorganic salts increases.

### 2.3. Effect of Elicitor Types and Concentrations on Growth and Metabolic Change in Adventitious Roots

**Figure 4:**
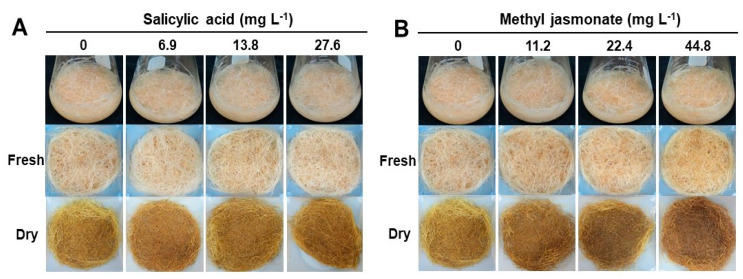
Effect of salicylic acid (**A**) and methyl jasmonate (**B**) concentrations on adventitious root growth of *A. macrocephala* after 40 days of culture on MS medium containing 5 mg L^−1^ IBA.

**Figure 5:**
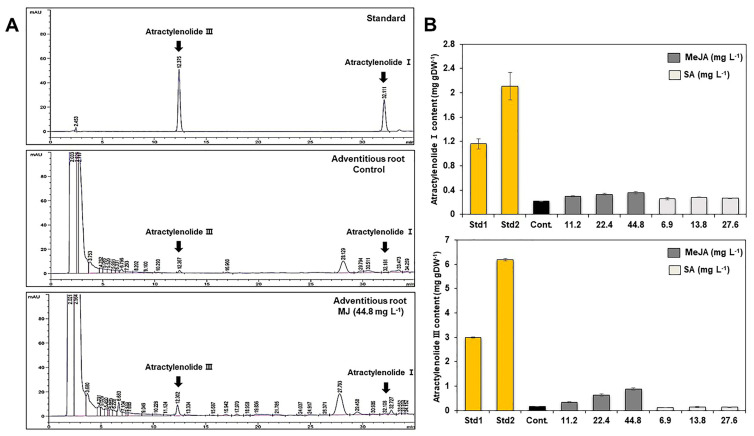
Typical HPLC chromatograms (**A**) and quantification of atractylenolide I and III (**B**) from adventitious roots of *A. macrocephala* after elicitor treatments. Std1, Std2: standard medicinal parts. Cont.: non-treated elicitors of adventitious roots.

The effect of elicitor types and concentrations on the growth of adventitious roots was examined (Figure 4). To investigate the effect of elicitor types and concentrations on the growth of adventitious roots, the roots were transferred to MS medium containing several concentrations of salicylic acid (SA) and methyl jasmonate (MeJA) during the last week of the whole growth process. After one additional week of incubation, the change in the growth of the adventitious roots was examined. In SA treatments, there was no significant difference in growth rates of adventitious roots regardless of concentrations (Figure 4A, see Supplementary Materials, Figure S4A,C). Similar to SA treatments, there was no significant difference in growth rates of adventitious roots from MeJA treatments (Figure 4B, see Supplementary Materials, Figure S4B,D). However, the scent of adventitious roots became stronger, and their color changed to dark brown in the 44.8 mg L^−1^ MeJA treatment. To quantify the atractylenolide I and III contents from adventitious roots, HPLC analysis was conducted (Figure 5, see Supplementary Materials, Figure S5A,B). Elicitor (MeJA, SA)-treated adventitious root samples showed a peak at the retention time of 12.375 (atractylenolide III) and 32.111 (atractylenolide I) on the HPLC chromatogram (Figure 5A). In the 44.8 mg L^−1^ MeJA treatment, the atractylenolide I content was the highest at 0.35 mg g^−1^, and it was 1.7 times higher than that of the control (0.21 mg g^−1^). The contents of Atractylenolide I and III were the highest at 0.87 mg g^−1^ in the 44.8 mg L^−1^ MeJA treatment, which was 5.8 times higher than that of the control treatment (0.15 mg g^−1^). In the case of the MeJA treatment, the atractylenolide content increased as the MeJA concentration increased. However, the SA treatment did not significantly affect the change in the contents of atractylenolide I and III in adventitious roots of *A. macrocephala,* even though the concentration increased (Figure 5B). To check whether the adventitious root could be used as an alternative means for standard medicinal parts of *A. macrocephala*, the content of atractylenolides I and III from two standard samples (Std1 and Std2) was also investigated (see Supplementary Materials, Table S1). The content of atractylenolide I from Std1 and Std2 was 1.16 ± 0.08 and 2.1 ± 0.23, and atractylenolide III was 2.99 ± 0.03 and 6.19 ± 0.05, respectively. These results clearly show that the content of atractylenolides I and III from adventitious roots was much lower than those of two standard samples, even though elicitation treatments has been conducted. However, the contents of atractylenolide I and III was different by 1.8 to 2 times between standard samples Std1 and Std2.

**Figure 6:**
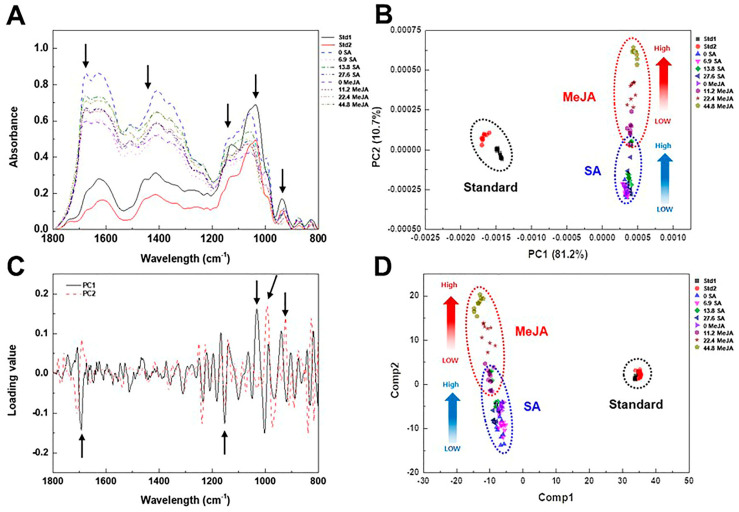
Multivariate analysis of FT-IR spectral data from cell extracts of standard rhizome parts and elicitor-treated adventitious roots of *A. macrocephala*. (**A**) Comparison of FT-IR spectral data from adventitious roots and standard rhizome parts. (**B**) PCA score plot of the FT-IR spectral data from adventitious roots and standard rhizome parts. (**C**) PCA loading plot based on PCA data from adventitious roots and standard rhizome parts. (**D**) PLS-DA score plot of FT-IR spectral data from adventitious roots and standard rhizome parts. Arrows indicate the FT-IR spectral regions showing significant variations between spectral data (**A**,**C**). Dotted circles represent each cluster for each elicitor treatment and standard rhizome parts (**B**,**D**).

To investigate the effect of elicitor types and concentrations on the metabolic change in adventitious roots, whole cell extracts from elicitor-treated adventitious roots were subjected to FT-IR spectroscopy (Figure 6). Similar to the FT-IR analysis of IBA concentrations, major spectral changes from elicitor-treated adventitious roots were observed in the 1700–1500, 1450–1200, and 1100–900 cm^−1^ regions of FT-IR spectra (Figure 6A). The PC loading plot indicated the major spectral variations that have an important role in the discrete clustering pattern on the PCA score plot were the 1700–1500, 1250–1100, and 1100–900 cm^−1^ regions of FT-IR spectra (Figure 6C).

The PCA and PLS-DA score plot also showed that adventitious roots and the standard medicinal parts were clearly discriminated (Figure 6B,D). The PCA score plot showed that PC1 and PC2 accounted for 81.2% and 10.7% of the total variation, respectively (Figure 6B). The replicated samples from each treatment were grouped in discrete clusters on the PCA score plot. The PC1 axis of the PCA score plot showed a separation pattern between adventitious roots and the standard medicinal parts, but the PC2 axis showed a discrete separation pattern into two groups corresponding to SA and MeJA treatments (Figure 6B). Similar to the PCA score plot, adventitious roots and the standard medicinal parts were clearly discriminated on the PLS-DA score plot (Figure 6D). Furthermore, the whole metabolite pattern from adventitious roots within SA and MeJA treatments changed as the concentration of elicitor increased. These results clearly suggest that there is a significant difference between adventitious roots and standard medicinal parts at the whole metabolite level.

## 3. Discussion

Adventitious roots proliferated in in vitro culture conditions that contained suitable phytohormones and produced important secondary metabolites [25]. Thus, the research established the optimal culture conditions for mass proliferation of adventitious roots of *A. macrocephala*. To achieve this goal, the effect of several environmental conditions in the culture, including IBA concentrations, types of media, inorganic salt concentrations of MS medium, and elicitor types and concentrations on adventitious root growth was examined.

The exogenous supply of plant growth regulators are required not only for the growth of adventitious roots, but also for the induction of in vitro morphogenesis. Auxin and ethylene act as the primary activators, while cytokinin and ethylene play roles primarily as inhibitors [26]. Many studies have also reported that the efficacy of diverse auxins for the establishment and propagation of in vitro grown adventitious roots varies according to the family and the species of the plant [27]. IBA is the most suitable effective auxin for the induction and development of adventitious root culture of medicinal plants [25]. Many studies report that IBA had a stimulatory role in root growth. Moreover, the optimal IBA concentration was different from species to species. Low concentrations of IBA (less than 2 mg L^−1^) are more effective for adventitious root growth of *Couroupita guianensis* [28] and *Hypericum perforatum* [29]. High concentration IBA treatment (more than 3 mg L^−1^) was effective for adventitious root growth in some plants, including *Cynanchum wilfordii* [21], *Orthosiphon stamineus* [30], *Panax quinquefolius* [31], and *Panax ginseng* [32]. In this study, the adventitious roots of *A. macrocephala* proliferated more rapidly at a high IBA concentration (5–10 mg L^−1^) than at 1 mg L^−1^ IBA or less (Figure 1).

In general, media properties such as media type and inorganic salt concentrations have an impact on the induction and proliferation of adventitious root culture of medicinal plants. The choice of culture media for establishment and growth of adventitious roots in medicinal plants is determined by the species of the plant itself [25]. It has been reported that MS medium was highly suitable for the proliferation of adventitious roots of *Rumex crispus* [33], *Echinacea purpurea* [34], and *Andrographis paniculata* [35]. In the present study, the adventitious roots of *A. macrocephala* proliferated more rapidly in MS medium than in SH and B5 media (Figure 2). However, Kim et al. [36] insisted that B5 medium was much better than any other culture media for adventitious root cultures of *Scopolia parviflora*. In addition, the inorganic salt concentration in the MS medium also affects adventitious root growth. It has been reported that the full strength MS medium was suitable for the proliferation of adventitious roots in *Rumex crispus* [33] and *Oplopanax elatus* [37], while the half-strength MS medium was optimal for *Echinacea purpurea* [34] and *Echinacea angustifolia* [38]. This study found that the adventitious roots of *A. macrocephala* proliferated more rapidly in higher concentrations (1 and 2×) of MS inorganic salt medium rather than lower concentration treatments (Figure 3).

Among the various types of abiotic elicitors, MeJA was the most representative chemical elicitor for adventitious root cultures of medicinal plants such as *Hypericum perforatum*, *Panax ginseng*, *Polygonum multiflorum*, and *Scopolia parviflora* [39,40,41,42]. MeJA can increase the synthesis of secondary metabolites in adventitious root cultures through signal transduction which accelerates the enzyme catalysis process, thus forming bioactive compounds such as alkaloids, flavonoids, terpenoids, and polyphenols [43]. Similar to MeJA, SA also has the potential to increase secondary metabolite accumulation in the adventitious root culture of medicinal plants. Furthermore, SA is a plant hormone that can stimulate the expression of the biosynthetic pathway genes of secondary metabolites. Moreover, SA and MeJA can activate the defense mechanism of plants. Thus, SA and MeJA can have an adverse effect on the growth of adventitious roots in increased concentrations [41,42]. There was no significant difference in growth rates in adventitious roots in SA treatments regardless of the concentrations (Figure 4A, see Supplementary Materials, Figure S4A,C). Similar to SA treatments, there was no significant difference in growth rates of adventitious roots in MeJA treatments (Figure 4B, see Supplementary Materials, Figure S4B,D). The SA and MeJA treatment did not significantly affect the growth of adventitious roots of *A. macrocephala* because the SA and MeJA treatment was performed after the proliferation of adventitious roots of *A. macrocephala*. In addition, the SA and MeJA treatments were performed for only one week in this study. Thus, it is assumed that the short treatment period did not affect the proliferation of adventitious growth.

To investigate the effect of elicitor types and the concentrations on metabolic change in adventitious roots, whole cell extracts from elicitor-treated adventitious roots were subjected to FT-IR spectroscopy (Figure 6). There have been many previous studies of FT-IR peak assignment and chemical composition. Spectroscopic techniques yield spectra that contain key bands that are characteristic of individual components. The data provide information about the chemical composition of the sample, including both primary and secondary metabolites [44,45]. Carbohydrates, including cellulosic fibers, monosaccharides, and polysaccharides, give a complex fingerprint in the characteristic bands visible in the 900–1200 cm^−1^ region of the infrared spectrum. Cellular proteins and amino acids give characteristic peaks of 1500–1700 cm^−1^ and 1200–1500 cm^−1^, that are designated as amide I, amide II, and amide III, respectively [46]. In this study, FT-IR combined with multivariate analysis was capable of discerning metabolite differences between adventitious roots and the standard medicinal parts of *A. macrocephala*. The greatest variation observed was in the carbohydrate, amide, and phospholipid/DNA/RNA regions (900–1200, 1500–1700, and 1300–1500 cm^−1^, respectively) of FT-IR (Figure 6). These results indicate that qualitative and quantitative metabolic changes corresponding to the polysaccharide, protein amide I and II, and polyphenol regions were important for discrimination between adventitious roots and the standard medicinal parts of *A. macrocephala*.

In this study, the content of atractylenolide I from the adventitious root was 5.2–9.5 times lower than that of Std1 and Std2. In addition, the content of atractylenolide III was also 19–39 times lower than that of Std1 and Std2 (see Supplementary Materials, Table S1). However, the contents of atractylenolide I and III from two standard samples changed depending on different collection areas. The content of metabolites from plant samples can be easily changed by modifications in physicochemical conditions such as cultivation period and areas, soil conditions, or environmental conditions. Unfortunately, the content of atractylenolide I and III from adventitious roots was insufficiently low compared to the standard medicinal parts. In this study, we firstly examined the tissue cultural factors related to the mass proliferation of adventitious roots except for chemical elicitation. Thus, it is inferred that those tissue cultural factors may not significantly affect the change in the productivity of secondary metabolites from adventitious roots of *A. macrocephala*. Moreover, the whole incubation period of the adventitious roots was only 40 days. Therefore, if the culture period of adventitious roots is fully extended or the physical conditions such as culture temperature and light conditions are changed, it is expected that the productivity of atractylenolide from the adventitious roots can be increased. Furthermore, FT-IR analysis showed that overall metabolite content, including polysaccharide, protein, and polyphenol from adventitious roots, was significantly different from that of the standard medicinal parts (Figure 6). In the future, we are going to analyze various physiological activities, including antioxidant, anti-bacterial, and cosmetic activity, using adventitious roots of *A. macrocephala*. These studies will clarify whether the adventitious roots could be used as an alternative means for standard medicinal parts of *A. macrocephala*.

The present study evaluated the impact of several cultural environment factors, including IBA concentration, media types, MS inorganic salt concentrations, and elicitor types and concentrations, on the establishment of the in vitro proliferation system of adventitious roots. Furthermore, the study also revealed the metabolic discrimination system between adventitious roots and the standard medicinal parts by FT-IR, combined with multivariate analysis after the application of several cultural environment factors and elicitors. This research is critical because current agriculture demands a new approach that can overcome the harmful factors affecting the cultivation of crops such as biological or abiotic stress, pesticides, heavy metals, and poor soil conditions. The impact of the present study suggests an alternative form of cellular agriculture for the production of biomass and bioactive compounds employing *A. macrocephala*.

## 4. Materials and Methods

### 4.1. Plant Materials and Induction of Adventitious Roots from Leaf Explants

The in vitro shoots of *A. macrocephala* Koidz. were provided by Dr. Jae Whune Kim of the Plant Biotechnology Park (9-4, Hari-gil, Bokheung-myeon, Sunchang-gun, Jeollabuk-do 595-833, Republic of Korea). The shoots were subcultured onto MS basal medium at four-week intervals. The basal medium consisted of MS salts and the pH was adjusted to 5.8 using 1N NaOH, and then it was sterilized at 121 °C for 15 min. To induce adventitious roots, root explants were cut into small segments (approximately 1 cm in length). The root explants were then cultured onto a MS medium supplemented with 1 mg L^−1^ indole-3-butyric acid (IBA), 30 g/L sucrose, and 0.6% Phyto agar at 25 °C in the dark. After six weeks of incubation, the adventitious roots derived from root explants were cut and subcultured onto the same fresh medium every 4 weeks for a total of 2 months. Unless stated otherwise, all culture media contained 30 g L^−1^ sucrose and 0.6% Phyto agar. For liquid media, Phyto agar was omitted. All cultures were maintained in the dark at 25 °C.

To establish the suspension culture system of adventitious roots, roots were cut into small segments (approximately 2 cm in length) and then transferred to a 250 mL Erlenmeyer flask containing 50 mL of liquid culture medium of the same composition. Suspension culture of adventitious roots was maintained in a shaking incubator (JSSI-300CL, JSR, Republic of Korea) at 100 rpm agitation at 25 °C in the dark.

### 4.2. Effect of IBA Concentrations, Types of Culture Media, and Inorganic Salt Concentrations in Culture Medium on Growth of Adventitious Roots

To accelerate the proliferation of adventitious roots in the suspension culture system, the effect of IBA concentrations, types of culture media, and inorganic salt concentrations in the culture media on the amount of fresh and dry weight in adventitious roots was examined. Approximately 30 mg (fresh weight) of suspension cultured adventitious roots were transferred to an Erlenmeyer flask (100 mL) containing 25 mL of liquid culture medium, supplemented with 30 g L^−1^ sucrose and 0, 0.1, 0.5, 1, 5, and 10 mg L^−1^ IBA, respectively. The adventitious roots were collected and washed with distilled water every 10 days in the culture. After removing sufficient moisture from the surface, the fresh weight was measured and then the roots were dried for 48 h in a dry oven at 60 °C so as to measure the dry weight of adventitious roots. The changes in fresh weight and dry weight of adventitious roots were investigated for 40 days of the culture. All treatments were repeated 3 times. Adventitious root samples from each treatment were also freeze-dried and pulverized into fine powder, using a pestle and mortar, for FT-IR analysis.

To examine the effect of culture media on the proliferation of adventitious roots, suspension-cultured roots were transferred to liquid MS, SH, and B5 media that were supplemented with 5 mg L^−1^ IBA and 30 g L^−1^ sucrose. All culture conditions, inoculation methods, and the measurements of fresh and dry weight were performed in accord with the above procedures.

The effect of inorganic salts in the culture medium on growth of adventitious roots was also examined in the same manner. The suspension cultured roots were transferred to solutions with 1/4, 1/2, 1, and 2 times the strength of MS liquid medium, supplemented with 5 mg L^−1^ IBA and 30 g L^−1^ sucrose. All culture conditions, inoculation methods, and measurements of fresh and dry weight were performed in accord with the above procedures.

### 4.3. Effect of Elicitor Types and Concentrations on Growth and Metabolic Change in Adventitious Roots

To investigate the effect of elicitor types and concentrations on metabolic change in adventitious roots, each elicitor was treated during the last week of the whole growth process. The concentrations of salicylic acid (SA) and methyl jasmonate (MeJA) treatments were adjusted to 0, 6.9, 13.8, and 27.6 mg L^−1^, and 0, 11.2, 22.4, and 44.8 mg L^−1^, respectively. After one additional week in the culture, adventitious roots were collected for examination of their fresh weight and dry weight. All culture conditions, inoculation methods, and measurements of fresh and dry weight were performed in accord with the above procedures. All treatments were repeated 3 times. Adventitious root samples collected from each treatment were also freeze-dried and pulverized into fine powder using a pestle and mortar for HPLC and FT-IR analysis.

### 4.4. Quantification of Atractylenolide from Adventitious Roots by HPLC Analysis

Before analysis, the freeze-dried standard medicinal parts and adventitious roots treated with elicitors of *A. macrocephala* were pulverized, powdered, and stored at −70 °C. The medicinal parts, two standard samples (Std1 and Std2), were collected from A and B, which are the representative production areas of *A. macrocephala* in the Republic of Korea. These medicinal parts were kindly provided by the Korea Institute of Oriental Medicine, and each elicitor-treated lyophilized adventitial root and standard medicinal parts were quantified using HPLC for quantitative analysis of atractylenolide I and III. Each one gram of root powder was extracted into 95% ethanol (1 g × 30 mL) for 4 h using an ultrasonic extractor (Fisher Scientific Sonic Homogenizer Mod.60, Waltham, MA, USA) to ensure the complete extraction of bioactive compounds. All samples were centrifuged at 3000 rpm for 15 min and filtered through a 0.22 μm PTFE syringe filter. To obtain the extract, the ethanol solution was evaporated in an oven at 40 °C, dissolved in sterile distilled water, and then freeze-dried. The standard solutions of atractylenolide I and III were prepared using 100% methanol (*v/v*). The extracts from adventitious roots were prepared with the same solvent used in the standard solution and analyzed using HPLC analysis. To construct a standard calibration curve, atractylenolide I and III solutions were prepared in methanol. The concentration to plot standard curves were 6.25, 12.5, 25, 50, and 100 µg mL^−1^ and high linearity of R2 > 0.999 was obtained from each calibration curve equation.

The HPLC analysis for quantification of atractylenolide I and III from elicitor-treated lyophilized adventitious roots and standard medicinal parts of *A. macrocephala* was performed using an Agilent 1200 series HPLC system (Agilent Technologies, Palo Alto, CA, USA), equipped with a diode array detector, binary gradient pump, autosampler, and vacuum degasser. The indicated compounds were eluted using a ZORBAX Eclipse Plus C18 column (C18, 4.6 mm I.D. × 250 mm, 5 µm particle size) at 25 °C with a flow rate of 1.0 mL/min. The injection volume was 10 µL with needle wash. The mobile phase comprised a mixture of acetonitrile and water. Atractylenolide I and III as standard compounds were eluted under gradient conditions (0–15 min, 50:50; 15–25 min, 60:40; and 25–35 min, 70:30). Data were collected and integrated using Agilent Chemstation B.04.01 software.

### 4.5. Whole Cell Extract Preparation for FT-IR Spectroscopy

To prepare crude whole cell extracts from root samples, 20 mg of powder derived from each root sample was mixed with 200 µL of 20% (*v/v*) methanol in a 1.5 mL Eppendorf tube, as described below. The mixtures were incubated in a 50 °C water bath for 20 min, followed by centrifugation at 13,000 rpm for 10 min. The resulting supernatants were then transferred to a fresh Eppendorf tube. These crude whole cell extracts obtained from adventitious roots and standard medicinal parts of *A. macrocephala* were stored at −20 °C until analysis by FT-IR spectroscopy.

### 4.6. FT-IR Spectroscopy and Multivariate Statistical Analysis

FT-IR spectroscopy analysis was performed according to the method of Kim et al. [17]. For the infrared measurements, a Tensor 27 FT-IR spectrometer (Bruker Optics GmbH, Ettlingen, Germany), equipped with a deuterated triglycine sulfate (DTGS) detector was used. After preheating the 384-well silicon plate to 37 °C, a supernatant sample (5 µL) was dropped on it and allowed to dry for 20 min. After drying the plate, it was placed in a microplate reader device (HTS-XT; Bruker Optics GbH, Ettlingen, Germany) and infrared spectra were obtained using the Tensor 27 spectrometer. Additionally, all FT-IR spectra were acquired with OPUS software (ver. 6.5, Bruker Optics Inc., Billerica, MA, USA). Infrared spectra were recorded from 4000 to 400 cm^−1^ using a spectral resolution of 4 cm^−1^ interval. A signal-to-noise ratio of 128 interferograms was co-added and averaged with the analytical results. The infrared spectra were obtained by subtracting the background spectra of the plate used for sample deposition. The original digital FT-IR spectra were collected from a spectral range of 1800 to 800 cm^−1^ for multivariate analysis. The pre-processing steps included baseline correction, spectral intensity normalization, and smoothing (ver. 6.5, Bruker Optics Inc.), as well as differentiation using Python software (version 2.7.15; Python Software Foundation). The processed spectral data were subjected to multivariate statistical analysis.

The FT-IR spectral data were imported into the R statistical analysis program (version 2.15.0; R Development Core Team) for the multivariate statistical analysis. The spectral data were limited to the 1800–800 cm^−1^ regions and then subjected to principal component analysis (PCA) and partial least square discriminant analysis (PLS-DA). PCA was performed using the non-linear iterative partial least squares (NIPALS) algorithm. The PCA loadings were then examined to identify the variables that were most important for discriminating between the root samples. To obtain a more distinct clustering of metabolic variation between the root samples, PLS-DA was applied.

### 4.7. Statistical Analysis

Statistical analysis between different groups was evaluated with a *t*-test. At least three biological replicates were performed for each analysis. Quantitative data are expressed as mean ± standard deviation (SD). A Student’s *t*-test was conducted in Excel.

## Data Availability

The data presented are contained within the article or Supplementary Materials.

## Supplementary Materials

The following supporting information can be downloaded at: https://www.mdpi.com/article/10.3390/plants12091821/s1. Table S1: Quantification of atractylenolide Ⅰ and Ⅲ from standard medicinal parts and adventitious roots with elicitor-treated of *A. macrocephala.* Figure S1: Multivariate analysis of FT-IR spectral data from cell extracts of adventitious roots and standard rhizome parts of *A. macrocephala* after incubation on different IBA concentrations; Figure S2: Multivariate analysis of FT-IR spectral data from cell extracts of adventitious roots and standard rhizome parts of *A. macrocephala* after incubation on different culture media; Figure S3: Multivariate analysis of FT-IR spectral data from cell extracts of adventitious roots and standard rhizome parts of *A. macrocephala* after incubation on different MS inorganic salt concentrations; Figure S4: Effect of salicylic acid concentrations on change of fresh weight (A) and dry weight (C) and methyl jasmonate concentrations on change of fresh weight (B) and dry weight (D) from adventitious roots of *A. macroscephala* after 40 days of culture on MS medium containing 5 mg L^−1^ IBA; Figure S5: Calibration curve used for atractylenolide I (A) and III (B) quantification by HPLC chromatography.
