# Supplementary material for: Metabolic Discrimination between Adventitious Roots and Standard Medicinal Part of Atractylodes macrocephala Koidz. Using FT-IR Spectroscopy
Source: Plants (Basel). 2023 Apr 28;12(9):1821. doi: 10.3390/plants12091821 (PMC10180665; doi:10.3390/plants12091821)
Supplement: Supplementary file 1 [file plants-12-01821-s001.zip › plants-2308455-supplementary.pdf]

Supplementary Materials

Supplementary Table S1 Quantification of atractylenolide Ⅰ and Ⅲ from standard medicinal parts and adventitious roots with elicitor-treated of *A. macrocephala*.

Treatment	Compound (mg g DW^{-1})	
	Atractylenolide Ⅰ	Atractylenolide Ⅲ
Std1	1.16 ± 0.08	2.999 ± 0.026
Std2	2.1 ± 0.23	6.193 ± 0.053
Cont.	0.219 ± 0.006	0.157 ± 0.001
MJ 11.2 mg L^{-1}	0.298 ± 0.012	0.34 ± 0.031
MJ 22.4 mg L^{-1}	0.331 ± 0.015	0.625 ± 0.045
MJ 44.8 mg L^{-1}	0.357 ± 0.016	0.876 ± 0.048
SA 6.9 mg L^{-1}	0.255 ± 0.019	0.121 ± 0.007
SA 13.8 mg L^{-1}	0.281 ± 0.006	0.141 ± 0.016
SA 27.6 mg L^{-1}	0.267 ± 0.002	0.128 ± 0.003

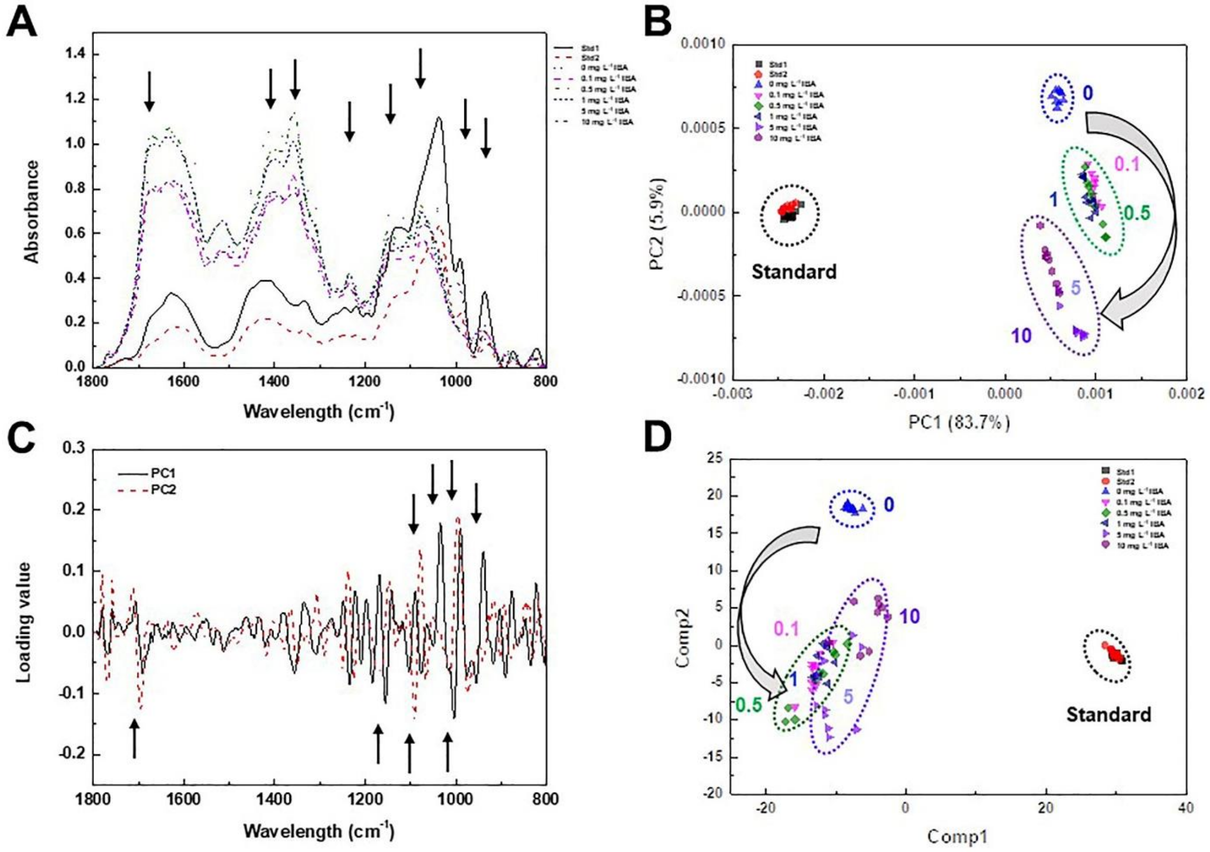

Supplementary Figure S1 Multivariate analysis of FT-IR spectral data from cell extracts of adventitious roots and standard rhizome parts of *A. macrosephala* after incubation on different IBA concentrations. A: Comparison of FT-IR spectral data from adventitious roots and standard rhizome parts. B**:** PCA score plot of the FT-IR spectral data from adventitious roots and standard rhizome parts. C: PCA loading plot based on PCA data from adventitious roots and standard rhizome parts. D: PLS-DA score plot of FT-IR spectral data from adventitious roots and standard rhizome parts. Arrows indicate the FT-IR spectral regions showing significant variations between spectral data (A, C). Dotted circles represent each cluster for IBA treatments and standard rhizome parts (B, D).

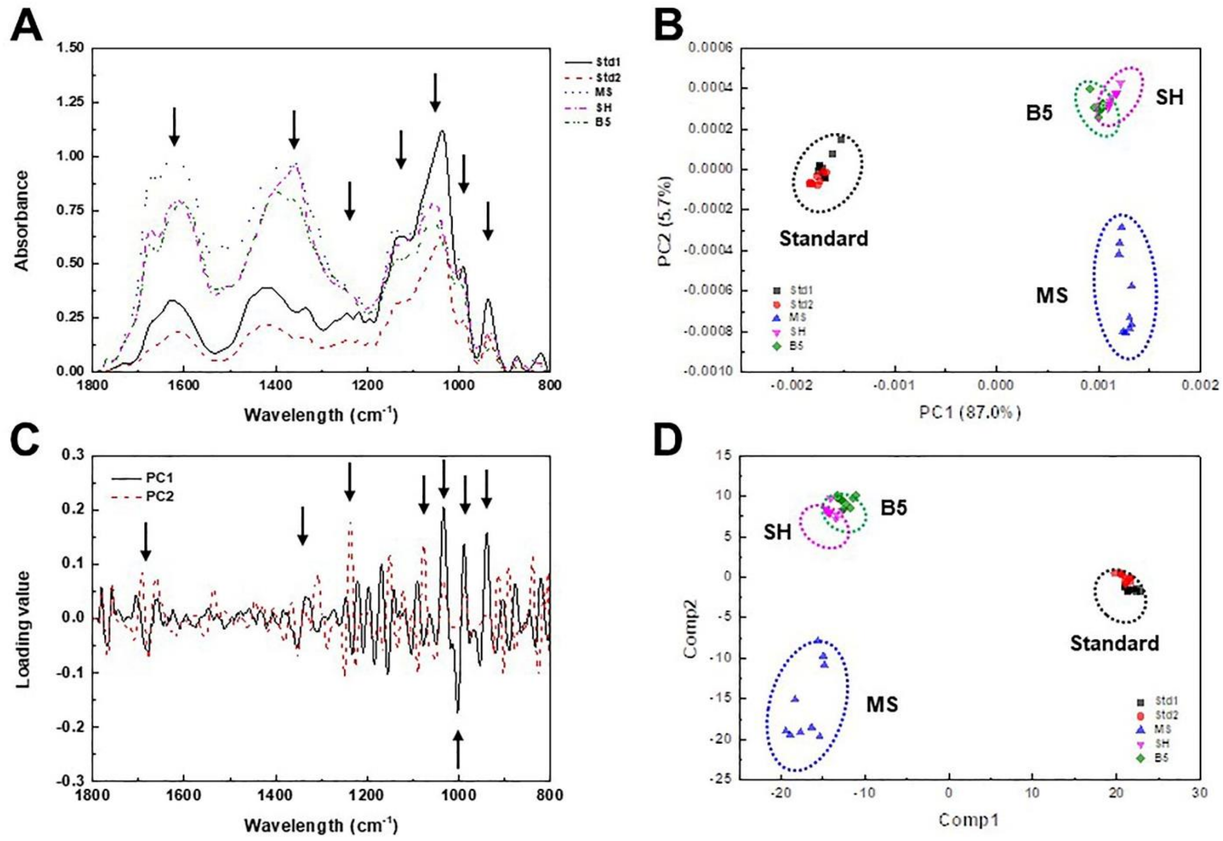

Supplementary Figure S2 Multivariate analysis of FT-IR spectral data from cell extracts of adventitious roots and standard rhizome parts of *A. macrosephala* after incubation on different culture media. A: Comparison of FT-IR spectral data from adventitious roots and standard rhizome parts. B**:** PCA score plot of the FT-IR spectral data from adventitious roots and standard rhizome parts. C: PCA loading plot based on PCA data from adventitious roots and standard rhizome parts. D: PLS-DA score plot of FT-IR spectral data from adventitious roots and standard rhizome parts. Arrows indicate the FT-IR spectral regions showing significant variations between spectral data (A, C). Dotted circles represent each cluster for culture media treatment and standard rhizome parts (B, D).

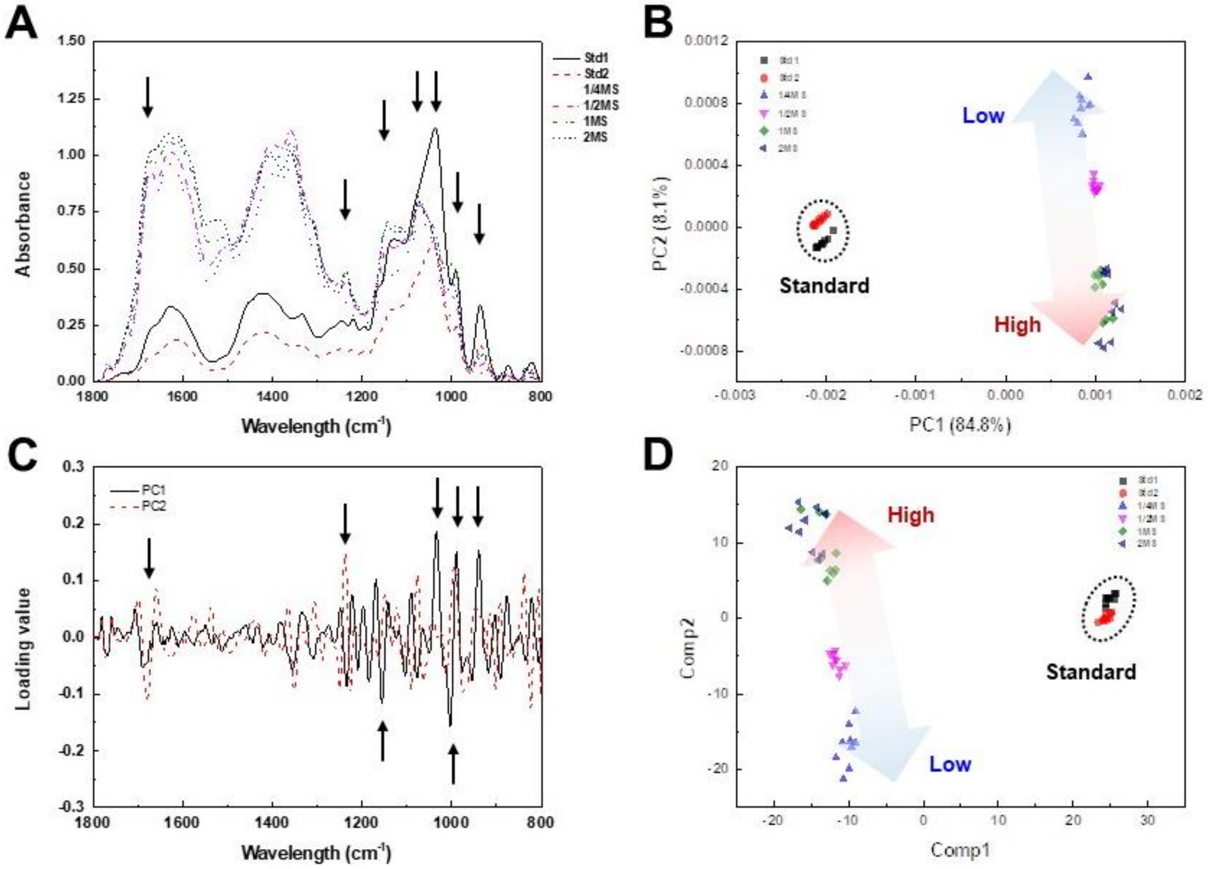

Supplementary Figure S3 Multivariate analysis of FT-IR spectral data from cell extracts of adventitious roots and standard rhizome parts of *A. macrosephala* after incubation on different MS inorganic salt concentrations. A: Comparison of FT-IR spectral data from adventitious roots and standard rhizome parts. B**:** PCA score plot of the FT-IR spectral data from adventitious roots and standard rhizome parts. C: PCA loading plot based on PCA data from adventitious roots and standard rhizome parts. D: PLS-DA score plot of FT-IR spectral data from adventitious roots and standard rhizome parts. Arrows indicate the FT-IR spectral regions showing significant variations between spectral data (A, C). Dotted circles represent standard rhizome parts (B, D).

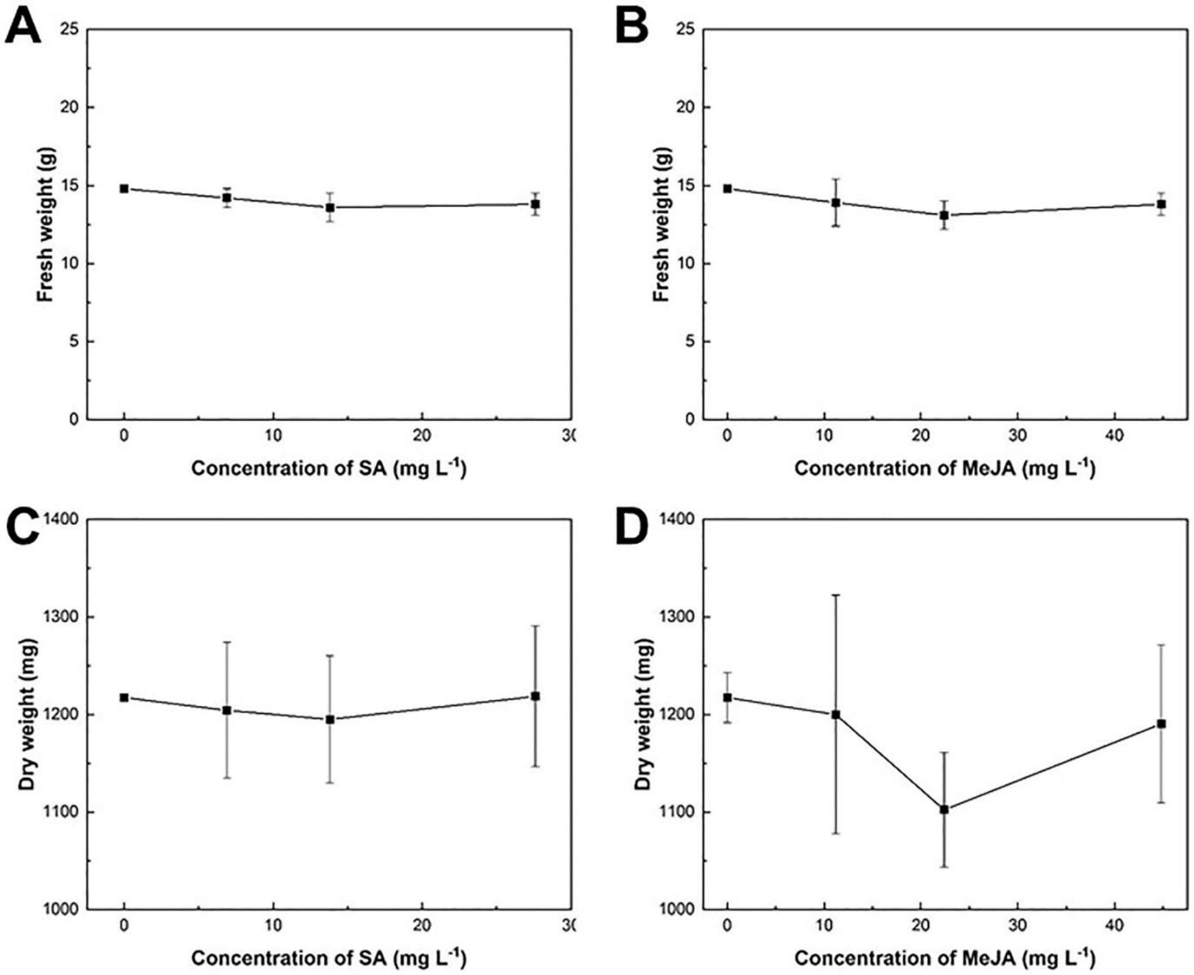

Supplementary Figure S4 Effect of salicylic acid concentrations on change of fresh weight (A) and dry weight (C) and methyl jasmonate concentrations on change of fresh weight (B) and dry weight (D) from adventitious roots of *A. macrosephala* after 40 days of culture on MS medium containing 5 mg/L IBA. Results are expressed as mean ± SD (n=3).

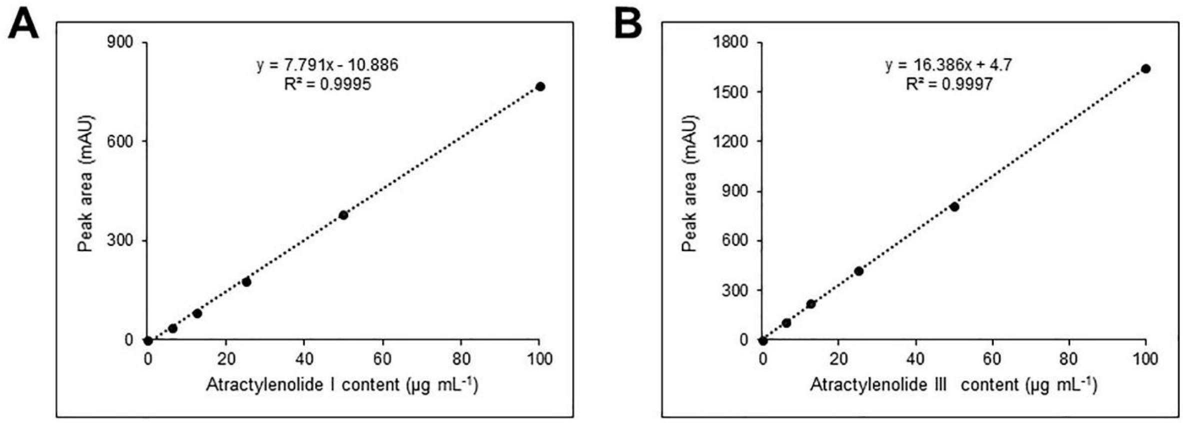

Supplementary Figure S5 Calibration curve used for atractylenolide Ⅰ (A) and Ⅲ (B) quantification by HPLC chromatography.
